# Retraction Note: MicroRNA-30d promotes angiogenesis and tumor growth via MYPT1/c-JUN/VEGFA pathway and predicts aggressive outcome in prostate cancer

**DOI:** 10.1186/s12943-023-01763-5

**Published:** 2023-03-20

**Authors:** Zhuo-yuan Lin, Guo Chen, Yan-qiong Zhang, Hui-chan He, Yu-xiang Liang, Jian-heng Ye, Ying-ke Liang, Ru-jun Mo, Jian-ming Lu, Yang-jia Zhuo, Yu Zheng, Fu-neng Jiang, Zhao-dong Han, Shu-lin Wu, Wei-de Zhong, Chin-Lee Wu

**Affiliations:** 1 Department of Urology, Guangdong Key Laboratory of Clinical Molecular, Medicine and Diagnostics, Guangzhou First People’s Hospital, Guangzhou, Medical University, Guangzhou, 510180 China; 2 Department of Urology, The Second Affiliated Hospital of Guangzhou Medical University, Guangzhou, Medical University, Guangzhou, 510260 China; 3 Institute of Chinese Materia Medica, China Academy of Chinese Medical Sciences, Beijing, 100700 China; 4 Guangdong Provincial Institute of Nephrology, Nanfang Hospital, Southern, Medical University, Guangzhou, 510515 China; 5 Urology Key Laboratory of Guangdong Province, The First Affiliated Hospital of Guangzhou Medical, University, Guangzhou Medical University, Guangzhou, 510230 China; 6 Graduate school of Jinan University, Guangzhou, 510632 China; 7 Department of Pathology, Massachusetts General Hospital and Harvard, Medical School, Boston, MA 02114 USA; 8 Department of Urology, Massachusetts General Hospital and Harvard Medical School, Boston, MA 02114 USA

**Retraction Note: Mol Cancer 16, 48 (2017)**

**https://doi.org/10.1186/s12943-017-0615-x**

The Editor-in-Chief has retracted this article. After a correction was published [1] to address concerns in a number of figures, additional concerns were raised, specifically:

- Figure 2a: there appears to be overlap between the miR-NC and the sh-NC panels
- Figure 2c: the sh-30d panel appears to be identical to the DU145 MYHPT1 panel of Supplementary figure 9c.
- Supplementary Figure 5D: the 0h panels for miR-NC and miR-30d appear to be identical.
- Supplementary Figure 6A: the sh-NC panel appears to overlap with the DU145 miR-30d+vector panel in figure 9a, but rotated.
- Supplementary Figure 9A: the LNCaP Vector panel appears to overlap with the DU145 miR-30d+MYPT1 panel.

The Editor-in-Chief therefore no longer have confidence in the results and conclusions of this article.

Author Wei-De Zhong has stated on behalf of all authors that they agree with this retraction.
